# Dr. Michéa on the Blood in the Neuroses

**Published:** 1848-07-01

**Authors:** 


### CHEMICAL INVESTIGATIONS ON THE BLOOD IN THE NEUROSES.

*A Memoir presented to the Academy of Sciences, at Paris, 29th November,* 1847, *by* Dr. Michéa.

The importance of knowing the relations that exist between derangements of the nervous system and the condition of the circulating system has long been fully admitted. Many authors, among whom we may instance Hoffman,[1] Alberti,[2] Wardenberg,[3] Dittmar,[4]

[1] Dissert. de mentis morbis ex morbosa sanguinis circulatione. Halle, 1700.
[2] Dissert. de commercio animæ cum sanguine. Halle, 1710.
[3] Dissert. de morbis animi ex anomaliis hæmorrhagicis. Halle, 1719.
[4] Dissert. de sanguinis et animæ nexu. Halle, 1744.

Bach,[5] Bergmann,[6] M. Brierre de Boismont,[7] Albers,[8] Juetting,[9] Vichoff,[10] and Friedreich,[11] have attempted to establish these relations with respect to neuroses affecting the mind. Their labours refer, however, solely to considerations drawn from pathological anatomy and clinical observations, or at most to a purely physical examination of the blood, whilst chemistry does not enter within the scope of their researches.

On the other hand, the chemical studies which have recently been undertaken on the blood in the neuroses are extremely incomplete, and relate either to too few cases, or to those in which the indications yielded do not appear to furnish all the guarantees necessary for the decision of a question of pure science.

In Germany, M. Hittorf has directed his attention to the examination of the blood of the insane, but his researches are limited to seven analyses, and refer solely to one grand division of insanity,—viz., *maniacal excitement.*[12]

M. Erlenmeyer, the author of a much more extensive work, establishes deductions, whilst he scarcely notices any of the chemical researches from which they have been educed. Of the 304 analyses which he says he has made, he instances only three, which relate to epilepsy complicated with mania.[13] Moreover, he has not examined the blood in simple cases, where the psychical affection was isolated and wholly independent of every kind of somatic lesion. He principally examined the blood in cases where madness existed conjointly with tuberculosis, organic affections of the heart or liver, pneumonia, dysentery, typhus, cancer, albuminuria, and other incidental diseases; so that his work, notwithstanding its importance and value with reference to pathological anatomy, affords but little aid in deciding the question at issue, since it leaves us in ignorance of the influence exercised on the condition of the blood by madness, which may, and frequently does, exist without producing any appreciable material disorder of the solids; whilst we are equally unable to distinguish whether the modifications experienced by the blood are the cause or effect of psychical disturbance, or whether they are the consequence of a somatic lesion—the symptom of an intercurrent disease.

In France there are no detailed works on the chemical analysis of the blood in the neuroses, with the exception of the researches of MM. Andral and Gavarret, in a case of facial tic douloureux;[14] of

[5] Abhandlung über die Schädlichkeit des allzu often Blutlassens in Ansicht der Seelenwirkung. Breslau, 1786.

[6] Ueber das Blut im Herzen. 1821.

[7] De la congestion cérébrale chez les aliénés. 1829.

[8] Bemerkungen über das Blutgefassystem bei Irren. 1830.

[9] Dissert de psychica sanguinis dignitate. Berol. 1830.

[10] Dissert de sanguinis congesti vi in vesania. Bonn, 1832.

[11] Handbuch der allgem. Pathologie der psychischen Krankheiten.

[12] Dissert. de Sanguine Maniacorum. 1846.

[13] Ueber das Blut der Irren (Archiv. für physiologische Heilkunde von Roser und Wunderlich. Stutgard, 1846. pp. 436 and 648.)

[14] Recherches sur les variations de proportion de quelques principes du sang. (Ann. de Phys. et de Chinie, t. lxxv. 2e série, p. 233.)

MM. Becquerel and Rodier, in a case of convulsions;[15] and of M. Emile Marchand, of St. Foy, in some cases of hysteria.[16]

The following is the table of the seven analyses of the blood of persons suffering from acute mania, made by M. Hittorf, in the Establishment for the Insane, at Siegburg:—

*Analysis of* 1000 *parts of blood.*

| | A girl aged 20. | A girl aged 23. | A married woman aged 37. | An unmarried woman aged 30. | A man aged 30. | A man aged 47, affected by periodical mania. | A man aged 48. |
|---|---|---|---|---|---|---|---|
| Water . . . | 794·559 | 803·884 | 805·701 | 803·345 | 788·999 | 779 093 | 751·995 |
| Fibrine . . | 2·083 | 1·932 | 3·173 | 1·929 | 1·396 | 1·870 | 1·455 |
| Globules . . | 109·191 | 116·967 | 119·576 | 112·010 | 118·199 | 137·898 | 130·808 |
| Albumen . . | 83·913 | 68·590 | 65·099 | 74·324 | 79·577 | 73·353 | 75·042 |
| Extractive matter, & soluble salts . . | 7·786 | 7·980 | 6·075 | 6·114 | 8·603 | 7·432 | 10·106 |
| Fat . . . | 2·468 | 0·647 | 0·376 | 2·278 | 3·226 | 0·354 | 0·594 |

From these seven analyses, the author has drawn the following conclusions:—

1. In acute mania, the blood does not undergo so great a change in its proportions as to lead *à priori* to the assumption of the fact.

2. The blood exhibits a diminution in the amount of the globules, and an increase in the amount of the water.

3. Mania is not the cause of the alteration in the proportion of these principles of the blood, that alteration being owing to the constitution of the patient.

4. This form of madness never exists conjointly with an acute plegmasia.

5. There is a difference between the blood of female maniacs and that of male maniacs.

The following are the three analyses of the blood made by M. Erlenmeyer, (at the Establishment for the Insane, at Prague,) on three patients, in two of whom mania was accompanied by epilepsy:—

Case 1.—Francis L., aged 66, of a strong constitution, robust, and plethoric habit, had several attacks of epistaxis. Three years before his entrance in the establishment, had suffered from repeated attacks of melancholy, in consequence of the loss of several thousand florins. From that time the attacks recurred every three or four months, and changed finally into a state of violent mania. The patient suffered from intense thirst, diarrhœa, and from cough accompanied by mucous expectoration; he died two months after his entrance into the Asylum for the Insane.

A *post-mortem* examination exhibited the following lesions: the face

[15] Recherches sur la composition du sang dans l'état de santé et de maladie. (Gaz. Méd. de Paris, 1844, No. 51, p. 815.)

[16] See a letter by this writer in the Gazette des Hôpitaux, March 6, 1847.

covered by reddish brown spots; the dura mater adhering to all points of the arch of the cranium, the internal surface of the membrane exhibiting sanguineous extravasations, whose superficies consisted of a rusty coloured organized membrane; the arachnoid presented, at the anterior portion, and at the base of the hemispheres, slight extravasations of blood organized in membranes on the left hemisphere; the pia-mater tolerably free from blood; softness of the convolutions; lateral ventricles dilated to twice their size, and containing each three drachms of clear serum; sanguineous extravasations in the anterior and medial fossæ of the cranium, especially at the left side; slight emphysema of the lungs; the inferior lobe, principally on the left side, turgescent, of a reddish gray tint, with a frothy œdema; red hepatisation at a great many points behind; surface of the kidneys atrophied and granular, &c. &c.

Blood was drawn some days before death.

*Analysis of* 1000 *parts of blood.*

| | |
|---|---|
| Water | 798·937 |
| Fibrin | 1·853 |
| Globules | 114·126 |
| Albumen | 73·635 |
| Salts and extractive matters | 10·820 |
| Fatty matters | 0·629 |

Case 2.—Charles G., aged 22, a weaver; his maternal grandfather died insane; had an aunt, also on the mother's side, who was subject to epilepsy, besides other relations who died at an early age of convulsions. At 14, he was himself seized with epilepsy. From the year 1843, the paroxysms of this neurosis have always been associated with a violent maniacal excitement, which either preceded or followed them. The patient was stout, and of a robust constitution.

Two ounces of blood were drawn during one of these attacks of maniacal excitement.

*Analysis of* 1000 *parts of blood.*

| | |
|---|---|
| Water | 815·747 |
| Fibrin | 2·301 |
| Globules | 105·596 |
| Albumen | 65·830 |
| Salts and extractive matters | 9·811 |
| Fatty matters | 0·715 |

Case 3.—Francis P., a shoemaker, aged 34. At the age of 16, in consequence of blows on his head inflicted by his master, he had attacks of epilepsy every fortnight, in the night; after a time they became more frequent, and ended in producing continual derangement of mind. Since the patient has been in the establishment, the attacks have only recurred three times in the year; the maniacal excitement is, however, very much more intense, being prolonged for several days after each attack. The patient is short and thick, florid, has blue eyes and black hair.

Two ounces of blood were drawn in the midst of a paroxysm of maniacal excitement.

*Analysis of* 1000 *parts of blood.*

| | |
|---|---|
| Water | 803·242 |
| Fibrin | 1·721 |
| Globules | 118·544 |
| Albumen | 67·325 |
| Salts and extractive matters | 8·530 |
| Fatty matters | 0·638 |

From these three analyses, and several others which he does not quote, M. Erlenmeyer draws the following conclusions:—

"The *venous* crasis (augmentation of the quantity of the globules) is very unusual in insane patients. It principally exists in idiocy and in *delirium tremens.*

"The *fibrinous* crasis (an augmentation of the amount of the fibrin) is likewise very rare in pure madness, that is to say, where the affection is free from all complications susceptible of modifying the proportions of this principle of the blood.

"There are two kinds of *serous* crasis, (diminution of the quantity of the globules, and increase in the proportion of the serum,) the one of which occurs with an augmentation of the mass of the blood, disposing to an exudation of the serosity by the kidneys and the serous membranes; and the other with a diminution of this mass, whose highest degree constitutes chlorosis. Of these, the latter, although in a less degree than in chlorosis, is the most commonly met with in the insane; it is manifested by a weak and quick pulse, *bruit de souffle* in the arteries, and a diminution of the quantity of the solid matters in the urine; being combined in most cases with cerebral plethora, which gives rise to considerable asthenia when combated by bloodletting.

"This second form of serous crasis principally occurs in mania and monomania, and becomes occasionally the cause of insanity."

In the blood of the patient suffering from facial tic douloureux, MM. Andral and Gavarret found nothing more than a slight augmentation of the quantity of the globules, (147·9).

In the case of convulsions already mentioned, MM. Becquerel and Rodier found that there was a considerable diminution of the globules (70), and an equally considerable deficiency of albumen, (43).

According to M. Emile Marchand, nervous diseases depend in general on, or are coincident with, a diminution of the globules of the blood. This diminution gives rise to *hysteria* in women, and to hypochondriasis in men. An augmentation of the globules (140, 150, 170, in the 1000) blunts the nervous sensibility, and induces apathy.[17]

The above is the historical exposition which I have given as an introduction to the present memoir, in order that the reader may be able to compare my researches on the subject under discussion with those made by other authors who have treated of this question.

My object will now be to make my readers acquainted with the results at which I have arrived from my own experiments on the changes in

[17] The work in which M. Emile Marchand gives these results has not yet been published. All that is known regarding it, reduces itself to some few vague assertions, wholly deficient in any kind of scientific demonstration.

proportion which may affect some of the principles of the blood; as, for instance, water, fibrin, the globules, and the solid matters of the serum, in the principal neuroses of intelligence, sensation, and motion. I will endeavour to verify the facts already established with relation to this subject, and at the same time attempt to fill up the numerous deficiencies left by the authors to whom I have already alluded.

Before we proceed to the consideration of the variations occurring in the proportions of the above-named principles of the blood in the neuroses, it is expedient that we should have a clear understanding of the composition of the blood in its physiological state.

According to M. Andral, for every 1000 parts of blood in the healthy subject, the mean value of the fibrin would be represented by 3, its minimum by 2·5, and its maximum by 3·5. The extremest minimum might be as low as 2, and the extremest maximum as high as 4, but such a proportion would only be of very rare occurrence, and dependent on idiosyncrasy. According to M. Nasse, the mean of fibrin is 2·5; according to M. Denis, 2·7; according to M. Lecanu, 2·9; and according to MM. Becquerel and Rodier, only 2·2.

According to MM. Andral and Gavarret, the mean of the globules is 127, the minimum 110, and the maximum 140. This last limit would, however, be associated with a plethoric condition. According to MM. Becquerel and Rodier there is a difference between the globules of women and of men. For the former, the mean of this principle of the blood is 127, the maximum 137, and the minimum 113; whilst in men the mean is 141, the maximum 151, and the minimum 131. My own analyses lead me to adopt the numbers given by MM. Andral and Gavarret in preference to those of MM. Becquerel and Rodier; and I am of opinion that they must have made choice of plethoric rather than healthy subjects, for in the case of an insane patient where there was evidently a considerable degree of cerebral congestion, to judge from the hemiplegia to which it gave rise, I found 148 for the globules.

According to the experiments of MM. Andral and Gavarret, the mean of water in the blood, when in its physiological state, is 790; that of the solids of the serum, 80, 72 parts of which are albumen, and the remaining 8 inorganic matters.

In order to approach as nearly as possible to a correct analytical knowledge of the blood in a pathological state, we must take into account certain physiological conditions, and several purely incidental circumstances, by which the elements of the blood may be modified, since without such a precaution we might be led into the error of ascribing to the disease changes which rather depend on the conditions and circumstances already named. The influences of this nature which would tend to affect the means of these numbers, either by raising them towards their maximum, or lowering them towards their minimum, are, 1. temperament; 2. constitution; 3. age; 4. sex; 5. food; 6. spontaneous, or artificially induced sanguineous discharges; and, 7. the state of the cutaneous, intestinal, and pulmonary secretions.

1. According to M. Lecanu, the effect of the lymphatic temperament is to diminish the quantity of the globules, whilst the sanguineous temperament tends, on the contrary, to raise the number of this principle of the blood. The nervous temperament is said to exercise the same influence on the globules as the lymphatic, although in the case of the former the action is alone constant and necessary.

2. According to M. Andral, the strength of the constitution exercises the most marked influence in raising the globules to their maximum, whilst hereditary or acquired weakness lowers them towards the minimum. MM. Becquerel and Rodier, who partially adopt these views, are of opinion that weakness of constitution likewise diminishes the quantity of the albumen, although the action it exercises on that constituent is less marked than on the quantity of the globules.

3. M. Denis supposes the globules to diminish after the fortieth year; whilst MM. Becquerel and Rodier are of opinion that the blood, between the ages of 50 and 66, differs but little from its condition at the age of maturity. The only change would probably be a diminution of the fibrin, although the mean representing this change would not exceed 2.

4. M. Lecanu was the first to establish the fact that the globules occur in smaller quantities in the blood of women than in that of men. His views coincide in this respect with those of Becquerel and Rodier. I must, however, observe, with respect to these two authors, that they have placed the mean, the maximum, and the minimum of the globules in the blood of men so much too high, that without reducing their numbers very considerably, we should be led into the error of mistaking a morbid for a physiological condition.

5. Diet, a diminution of food, or any disturbance in the process of chymification, has the effect of perceptibly reducing the proportion of the globules, exercising but a very small action on the other principles generally; as, for instance, on the albumen. On the other hand, we are led from analogy to conclude that too great an abundance of food, and too great activity in the functions of digestion and assimilation, must tend to modify the globules and the other elements, in accordance with a relation identical in its nature, but opposite in its direction.

6. Artificially induced sanguineous discharges always diminish the quantity of the globules, and in a far less degree the proportions of the fibrin and albumen. They appear to reduce these numbers in a ratio corresponding with their frequency and approximation. It must, however, be observed, that, according to the experiments and analyses of MM. Andral and Gavarret, the globules do not diminish in the same proportion in all individuals, from the first to the second or third period of venesection, and that these circumstances give rise to great differences in individual cases. Metrorrhagia, or any other kind of spontaneous sanguineous discharge, acts in the same manner as bloodletting.

7. Finally: perspiration, transpiration, expectoration, or too abundant evacuations, tend likewise to lower the proportion of the globules.

It will, of course, be understood, that in all these conditions of the economy, the number representing water is high in proportion to the low degree of that of the other principles of the blood.

The greater part of my researches, with reference to madness and epilepsy, were made at the Bicêtre, in the wards of Merse, Leuret, Voisin, Moreau, and Delasiauve; and while I feel that I can offer but a slight tribute of gratitude on the present occasion to these valued teachers and colleagues, I cannot wholly pass over the extreme kindness and promptitude with which they furnished the materials necessary for the present memoir. I have likewise made several experiments on the blood of patients residing in a private establishment in Paris, which is principally designed for the reception of the insane, and of persons suffering from nervous affections.

The following method was the one I adopted in separating the principles of the blood which I wished to examine:—

Immediately after the blood was drawn, and whilst it was still warm, I divided it into two equal parts, in the following manner:—I poured the first and the fourth parts of the blood drawn into one vessel, and the second and third portions into another vessel; these vessels were then completely filled, and closed with ground-glass stoppers, in order that the filaments of the fibrin might not be separated, or any portion of the aqueous element lost by evaporation, during their transit from the hospital to the laboratory.

The first portion of the blood was weighed in a porcelain capsule immediately after its removal from the closed vessel in which it had been conveyed. After being dried on a stove, it was again weighed; the difference of the results yielded by the two processes of weighing represented the quantity of the water. The second portion was allowed spontaneously to coagulate. The serum was carefully separated from the clot by means of a pipette, and was set aside after having been dried in a water-bath. The clot that had been removed from the serum was tied up in a piece of very fine linen, and was then digested in seventy times its weight of distilled water.

By this process of digestion, the fibrin was found in the linen, and after being washed, was dried and weighed. The water in which the clot had been washed, was raised to and kept at the temperature of 150° Fahrenheit to 164° Fahrenheit, until it was completely decolorized. When it has been thus deprived of its red colour, and the clots float in the midst of the grey liquid, the globules are coagulated.[18] The liquid was then poured on a filter, which suffered the water to pass, and retained the globules. The latter were first dried, and then weighed. Finally, the water in which these globules were suspended, and which was enclosed in the serous portion of the clot, was also evaporated to dryness, and the residue which it yielded, added to the residue of the evaporation of the serum, represented all the solid matters of this liquid. On treating them with boiling water, they were freed from the soluble salts and extractive matters contained in them; and after the complete

[18] The clot will always retain a certain quantity of the serum in its meshes, whatever measures we may adopt for the contrary. The experiments of M. Denis have shown that when albumen is associated with the globules in the proportion of one part of the first-named principle to twelve parts of the second, the globules will coagulate, and only carry away an extremely small quantity of albumen; so that on diluting the clot in seventy times its own weight of water, after having freed it as much as possible from the serum, and heating the liquid to 158° Fahrenheit, the globules may easily be obtained almost in a state of purity.

evaporation of the water, there remained only the organic matters, which were then dried and weighed.

The serum must be dried before a very gentle fire, in order to prevent its carbonization; it is known to be perfectly dried when it separates into small rounded portions, or granules, of an amber colour. These granules must then be carefully pulverized before they are weighed.

All the principles of the blood reduced to a state of complete dryness, must be weighed immediately on their being removed from the stove, since, without this precaution, an error may easily be made from the humidity which they absorb in cooling.

I would, finally, remark, that, in conformity with general practice, I have, by means of some slight calculation, reduced all the portions of the blood analyzed from the same bloodletting to 1000 parts.

This proceeding, which in most respects is analogous with that adopted by M. Denis, furnishes the ponderable values of the principles which I have endeavoured to extract from the blood. Moreover, from the peculiar circumstances in which I was placed, it was almost the only measure that I was able to adopt. In order, however, to escape a subject of reproach justly advanced against M. Denis, and to avoid the danger of obtaining too low a number for fibrin, I have taken the precaution of soaking the clot in a piece of closely woven linen, by means of which all the particles of the fibrin might be retained.

The researches to which I have devoted my attention have for their object two classes of consideration:—

I. Exclusively chemical facts.

II. Inductions relating to pathological physiology, and to the treatment of neuroses.

### SIMPLE OR COMPOUND DISEASES OF THE MIND.

My analyses have been made with reference to patients included in the following subdivisions of disease:—

1. Insanity accompanied by general paralysis.
2. Mania.
3. Monomania.
4. Insanity and stupidity.
5. Delirium tremens.
6. Idiocy or imbecility.

1. *Insanity accompanied by general paralysis.*—No chemical analysis has as yet been made in France, Germany, or England, of the blood of persons affected by this form of madness, the principal symptoms of which we will classify under several heads.

In the first period, at least *one half of the cases manifest ambitious or proud monomania.* The patient pretends that he is rich and powerful, possessed of dignities, distinctions, and honorary titles, and in the enjoyment of a multitude of other personal advantages. The countenance is generally red, and the features expanded, betraying the satisfaction and happiness experienced by the possessor of these imaginary riches and honours. At the same time, there is *very frequently* a diminution of tactile susceptibility or complete anæsthesia; almost always a weakening of the intellectual faculties, and a *constantly and habitually* manifested alteration in the movements. The weakness of the intellectual faculties affects the powers of the attention, which can no longer be easily fixed on any subject in particular, the judgment, which embraces only a small number of terms, and, lastly, the memory. The recollection of past events and of former ideas survives in part; but that of recent or passing events is nearly or wholly null. The alteration in the movements begins in a certain trembling of the lips, an embarrassment in the speech, manifested by a slowness, hesitation, or stammering in the pronunciation of certain words, and in a vacillation of the tongue. This disorder of phonation, which is occasionally very difficult of recognition at its origin, is followed by a slight difficulty in the manner of walking, scarcely to be detected unless the cause of the disease be suspected, and which is manifested by a trifling degree of stiffness in the limbs, an occasional stumbling, and a slight deviation from the straight line. This last symptom is, however, very frequently absent, especially in cases of excitement. Finally, towards the close of this period, the paralysis advances to the upper limbs—the fingers lose their force and suppleness; the patient employs a considerable time in buttoning his clothes, and in many cases even, is unable to effect his object.

In the second period, proud and ambitious monomania continues, accompanied by a condition of maniacal agitation, which varies from simple loquacity to the most decided fury. In the meantime, incoherence of ideas begins to be general. The patients pay no attention to what is passing around them, do not answer the questions proposed to them, or make extravagant replies. The embarrassment in the speech and the difficulty of moving the limbs increase, whilst the anasthesia, on the contrary, often diminishes, or entirely ceases.

The third period is generally characterized by complete insanity.

The understanding can only grasp a small number of vague ideas devoid of sequence. Proud and ambitious monomania often disappears, and the former agitation is replaced by calmness. Pronunciation becomes very difficult, and even occasionally unintelligible. The gait is extremely tottering, or walking and standing upright seem alike impossible. The excretions are involuntary, and the pharynx participates in the general paralysis.

With the exception of very rare cases, the pulse is natural in all three periods. The functions of nutrition exhibit no alteration. The patient retains his former embonpoint, the appetite is voracious; and far from diminishing with the progress of the disease, obesity and gluttony increase in proportion to the rapid advance towards death.

### FIRST PERIOD.

*Slight insanity—Absence of ambitious monomania—Incomplete general paralysis—Normal amount of the globules, solid matters of the serum, &c. &c.*

Case 1.—Fuet, a bailiff, aged 42, of a moderately strong constitution and sanguineous temperature, entered Bicêtre the 3rd of May 1847.

Confused pronunciation, hesitation in articulating certain words, inability to move the tongue without its vacillating. The patient can stand upright, or walk about, but the lower limbs seem insecure, and are moved with much caution and consideration. He even falls occasionally, when attempting to move faster; experiences some difficulty in using his upper limbs, dresses and undresses unassisted, although not without difficulty; the fingers tremble in attempting to button the clothes, &c. The sphincters of the anus and the bladder perform their functions.

This patient is wholly free from agitation, and exempt from every species of delirious hallucination; he does not suppose himself possessed of *titles, dignities, or fortune.* He makes correct and precise replies to the questions asked; asserts that his memory fails from day to day, although he remembers events that have occurred recently as well as those of older date. If it were not for a slight degree of insanity, a certain indolence, an absence of any prime motor in the mental activity, this patient might be considered sane.

Before his entrance at Bicêtre he had enjoyed a sufficiently generous diet, but his appetite was small. Constant apyrexia.

Two hundred grammes of blood were drawn from this patient on the 5th of May.

*Analysis of* 1000 *parts of blood.*

| | |
|---|---|
| Water | 783·67 |
| Globules | 125·02 |
| Fibrin | 2·56 |
| Solid matters of the serum | 88·75 |

*Cause unknown—Slight enfeeblement of the memory—Ambitious monomania—General incomplete paralysis—Absence of involuntary excretions—The proportions of the principles of the blood do not deviate from their normal physiological limits.*

CASE 2.—M. R., a merchant, aged 41, of a strong constitution. A cousin-german on his father's side was insane. Had never committed excesses of any kind; has never been exposed to any prolonged vexations, or violent grief. Six months ago presented a slight hesitation in his speech; made great exertions to articulate his words, pronouncing them with greater slowness and less precision. At the same time he manifested a partial derangement of mind, pretending to be possessed of an immense fortune, to be the head of some of the greatest mercantile houses of Paris, and to have money enough to purchase the whole of that city.

At the present time (May 18, 1846) M. R. presents the same aberration of mind; his memory is, however, almost wholly exempt from lesion of any kind. The hesitation in his speech is more decided, and is accompanied by difficulty in walking. The lower limbs appear to be somewhat stiff. He holds himself badly on his legs, places them apart, and when standing, does not appear to be completely in equilibrium; on attempting to hasten his steps he totters, and deviates from the straight line which he would follow. The upper limbs do not appear to be sensibly affected by his disorder. The actions of the bladder and rectum are performed with ease. The patient has an excessive appetite, which he satisfies by a copious and nourishing food. The constitution is not weakened by any hæmorrhoidal discharge, or any other excessive excretion.

*Analysis of* 1000 *parts of blood.*

| | |
|---|---|
| Water | 795·00 |
| Globules | 120·18 |
| Fibrin | 2·70 |
| Solid matters of the serum | 82·12 |

*Enfeeblement of the intellectual faculties—Absence of ambitious monomania properly so called, although there exists a feeling of self-satisfaction—General incomplete paralysis—Voracious appetite—Involuntary excretions—Diminution of the globules and of the fibrin—Augmentation of the water and the solid matters of the serum.*

Case 3.—Frances Q., aged 45, a baker, married; belongs to a family in which it is not known that any case of insanity has existed. She is of a florid complexion, and a sanguineous temperament.

Towards the close of July, 1846, this woman, without any apparent cause, moral or physical, experienced a weakness of memory, and a slight difficulty in the pronunciation of her words. These symptoms, instead of diminishing, or even remaining stationary, continued to increase.

On being placed in a private establishment for the insane, on the 1st of September, the patient was in the following state:—She is slow in understanding the questions addressed to her, which it is even necessary to repeat several times before she can comprehend their bearing. She presents no incoherence in her speech, answers correctly, and without hesitation, all questions regarding her age, birthplace, occupations, and family; but she loses the recollection of the persons or things whom she has seen recently, and especially forgets their names. She cannot recall the number of months that she has been absent from her family, and forgets one day the nature of the food she had taken on the preceding one.

She has no proud or ambitious monomania, does not believe herself possessed of titles, millions of money, or diamonds; but she is extremely contented and self-satisfied, smiles almost continually, declares herself happy in being well, and being beloved by her husband and children; is tolerably calm, and does not speak except when spoken to.

She expresses her ideas with hesitation and embarrassment, and has special difficulty in pronouncing certain words. She has convulsions in the muscles of the lips, and a tremor in the voice. Her limbs tremble, although not to such an extent as to prevent her using her hands, or walking; she even occasionally attempts to walk very fast, and almost to run, without falling or even tottering.

The functions of nutrition are in a healthy condition; her appetite is voracious. Menstruation has been somewhat more abundant than usual during the last two months; there is no paralysis of the bladder or rectum; no sanguineous discharges, no excess in the alvine evacuations, or in the perspiration.

448 grammes of blood (14 oz.) were drawn on the evening preceding her entrance in the establishment. On the 5th of September, five days afterwards, 192 grammes were again drawn; and from this blood I made the following analysis:—

*Analysis of* 1000 *parts of blood.*

| | |
|---|---|
| Water | 808·000 |
| Globules | 79·625 |
| Fibrin | 1·500 |
| Solid matters of the serum | 110 875 |

The diminution in the amount of the globules does not depend on the constitution of the patient, or on the food of which she partook, since she was very strong, and partly satisfied a very voracious appetite. It depends, in this case, on sex; also, in part, on somewhat too abundant a discharge of menstrual blood; and, lastly, on previous copious blood-letting, although the blood had time to recover from the effect of these losses during the five days which intervened before the second venesection from, which that portion was taken of which we have given an analysis.

*Insanity—Ambitious monomania—Diminution of tactile sensibility —Imperfect general paralysis—Absence of involuntary excretions —Very considerable augmentation of the globules—Diminution of the fibrin.*

CASE 4.—John Roumanille, a gardener, 40 years of age, of a strong constitution, a bilio-sanguineous temperament, and decided embonpoint. Entered Bicêtre in the month of May, 1847. On the 20th of July, he presented the following condition:—He replies correctly to questions asked regarding his Christian name, and the name of his family, his trade, the day of the month, &c., but he cannot recollect his age; says he is 60 years old; nor can he indicate the present year, nor state with any accuracy the date of his arrival at Bicêtre.

He has the most delirious ideas regarding his possessions and dignities; imagines himself to be colonel of the National Guard at Paris, Chevalier of the Legion of Honour, &c.; much embarrassment in pronunciation; walking and standing are alike difficult; trembling of the hands in attempting to button his clothes, which is effected very slowly; diminution of general sensibility; no involuntary excretions; habitual apyrexia; the appetite normal. The urine is not rendered turbid by heat, or on the addition of nitric acid. 300 grammes of blood were drawn.

*Analysis of* 1000 *parts of blood.*

| | |
|---|---|
| Water | 766·009 |
| Globules | 160·451 |
| Fibrin | 1·872 |
| Solid matters of the serum | 71·668 |

SECOND PERIOD.

*Insanity—Slight agitation—Absence of ambitious monomania—Diminution of tactile sensibility—Incomplete general paralysis—Voracious appetite—Attack of cerebral congestion—Augmentation of the globules.*

CASE 1.—Lef ......, artizan, aged 37, of a moderately strong constitution, and a bilio-sanguineous temperament. Has been ill only a short time. Entered Bicêtre on the 19th of June, 1847.

On the 24th, he presented the following condition :—Can neither indicate the day, month, nor year, but answers correctly all questions asked regarding his age, occupation, and family. He knows that he is at Bicêtre; insists earnestly on being discharged; weeps with great facility; has no delirious ideas of fortune or grandeur; a slight degree of agitation and loquacity; marked embarrassment in his speech; scarcely notable paralysis in the movement of his upper and lower limbs; considerable diminution of tactile sensibility; voracity; apyrexia; pulse 64; the urine is not rendered turbid by heat, or on the addition of nitric acid. 300 grammes of blood were drawn.

On the 25th, the patient was suddenly seized with a slight hemiplegia on the left side. 400 grammes of blood were again drawn.

On the 26th, the hemiplegia had entirely disappeared. The blood drawn on the first occasion was alone examined.

*Analysis of 1000 parts of blood.*

| | |
|---|---|
| Water | 770·879 |
| Globules | 155·000 |
| Fibrin | 2·476 |
| Solid matters of the serum | 71·645 |

The augmentation of the globules corresponds in this case with the vigour of the age of the patient, with the nature of his temperament, and the excess of the appetite, fully confirming the opinion of M. Andral, who regards this as one of the causes of cerebral congestion.

*Enfeeblement of the intellectual faculties—Self-satisfaction and contentment—Diminution of tactile sensibility—Imperfect general paralysis of motion—Attack of cerebral congestion—Augmentation of the globules.*

CASE 2.—Anthony Holland, hackney-coachman, aged 36; has been 18 months at Bicêtre. Of a strong constitution and a sanguineous temperament.

On the 15th of May, 1847, exhibited marked embarrassment in his speech. The patient is able to remain standing, but his lower limbs totter in walking; his hands continually tremble; and he is incessantly trying, to no purpose, to button his clothes. The sphincters of the anus and the bladder have lost none of their power of contracting; there is a diminution of tactile sensibility. The memory of this patient is much weakened; he can recall his own name, that of his birthplace, and even knows that he is at Bicêtre; but he cannot tell his age, nor the time at which he entered the establishment, &c. &c. He appears self-satisfied, smiles incessantly, thinks his hard hands beautiful, and regards his hospital-dress as magnificent. Nevertheless, he has no actual idea of possessing a fortune or dignities, &c. He eats without any very marked voracity.

On the 16th, this patient was suddenly seized with an attack of cerebral congestion; his face became purplish red; and he found it alike impossible to stand upright or to walk, &c. &c. Apyrexia: blood was drawn during this attack.

*Analysis of* 1000 *parts of blood.*

| | |
|---|---|
| Water | 769·00 |
| Globules | 148·60 |
| Fibrin | 2 62 |
| Solid matters of the serum | 79 78 |

The augmentation of the number of the globules coincides, in this case also, with the existence of cerebral congestion. It is further accounted for by the age of the patient, the nature of his temperament, and the strength of his constitution.

*Insanity—Ambitious monomania—Tactile sensibility entirely unimpaired—Imperfect general paralysis of motion—Attack of cerebral congestion — Augmentation of the globules — Diminution of the albumen.*

Case 3.—G. R——, an umbrella-man, aged 37. Of a moderately strong constitution, black hair and eyes, copper-coloured complexion, not very stout; of an irritable disposition. Entered Bicêtre in the beginning of April, 1847.

At the present time (1st of July, 1847) he presents the following condition:—Remembers with tolerable accuracy the food he has partaken of the preceding day; knows the month and the year, but is unable to state the date of the month. Believes he possesses castles at Paris, Vitry le Français, Aurillac, &c.; that the Tuileries belong to him; that they are made of gold, and covered with precious stones. He thinks that Napoleon is returned from St. Helena, and is now at the Tuileries, &c. Marked embarrassment in his speech; trembling of the upper limbs; walking and standing are alike attended by difficulty; occasionally convulsive shocks of the whole frame; the urine and fæces expelled voluntarily.

On the 2nd, the patient repeatedly fell down. Preserved his consciousness, but sensibility and mobility were notably diminished on the whole of the right side of the body; the face red, and the enunciation more embarrassed than usual. 500 grammes of blood were drawn by general venesection, and 200 grammes by cupping at the nape of the neck.

On the 3rd, the hemiplegia entirely disappeared; the tactile sensibility unimpaired in both sides of the body; the pulse rather soft—72 pulsations in the minute. 100 grammes of blood were again drawn. The urine not rendered turbid by heat, or on the addition of nitric acid.

The blood drawn at the second venesection was alone examined.

*Analysis of* 1000 *parts of blood.*

| | | |
|---|---|---|
| Water | | 800·412 |
| Globules | | 138·863 |
| Fibrin | | 3·254 |
| Solid matters of the serum | organic | 48·341 |
| | inorganic | 9·130 |

The number 138 does not exactly represent the quantity of the globules. It is evidently inferior to what it would have been if another very copious venesection (of 700 grammes) had not been made 24 hours previous to the second, which yielded the blood that has been examined. For this same reason, the number of the albumen is perhaps a little too small. Nevertheless, even if we make an allowance for this fact, the very marked diminution of this principle of the blood does not depend upon its loss by the urine, since the latter contains no vestige of it. There was neither œdema nor anasarca. Finally, the attack of cerebral congestion coincides, not with the diminution of the fibrin, but with the augmentation of the globules.

*Insanity—General paralysis of motion and feeling—Slight degree of proud and ambitious monomania—Voracious appetite—Excretions occasionally involuntary—Slight diminution of the globules, and very great diminution of the albumen.*

CASE 4.—Cof——, aged 37, a jeweller; has been eighteen months at Bicêtre. Has a good constitution, blue eyes, reddish hair, florid complexion, strong limbs, and presents considerable embonpoint. Continued trembling of the lips; decided embarrassment of pronunciation; tottering gait; a little trembling in the hands; occasional involuntary excretions; marked diminution of tactile sensibility; voracious hunger, which the patient attempts to satisfy by stealing the food of his neighbours; enfeeblement of memory; no recollection of things of recent occurrence; no very decided hallucination. Always replies that he is well, that he has laid by considerable savings, has fine clothes, and that his sister is married to a rich man.

On the 25th of May, 1847, his face became red, and he looked much excited; the pulse very full; no heat of the skin, (76 pulsations in a minute;) the urine was not rendered turbid by heat, or on the addition of nitric acid. 100 grammes of blood drawn.

*Analysis of* 1000 *parts of blood.*

| | | |
|---|---|---|
| Water | | 840·100 |
| Globules | | 108·970 |
| Fibrin | | 3·653 |
| Solid matters in the serum | organic | 40·000 |
| | inorganic | 6·677 |

Notwithstanding a strong constitution and a voracious appetite, the globules are somewhat below their physiological limit. Far from tending to produce a decoloration of the capillaries, this diminution, on the contrary, gave rise to redness and the plethoric appearance of the skin of the face. The enormous diminution of albumen appears to be wholly spontaneous, for the urine betrays no traces of this principle. There is an absence of serous infiltration in the cellular tissue, in the peritonæum, the pericardium, and the pleura.

*Enfeeblement of the intellectual faculties—Absence of ambitious monomania—General paralysis of motion—Diminution of sensibility—Augmentation of the globules—Diminution of the fibrin and albumen.*

CASE 5.—V——, a cook, aged 55, of a vigorous constitution, and a bilio-sanguineous temperament. Entered Bicêtre in 1844.

At the present time, (July 14, 1847,) his intellectual faculties are much impaired; he recollects his name and age, but he does not know where he is, or the day of the month and year. He often exhibits incoherence in his ideas, although he has no hallucinations regarding fortune or greatness.

His pronunciation is much embarrassed. The trembling of the muscles presiding over phonation is so great, that his words are scarcely intelligible, and are not articulated. In some instances, articulation is impossible; the muscles of the pharynx are occasionally unable to convey the food into the œsophagus; and it is even found necessary in these cases to remove the food lodging in the buccal cavity, in order to prevent the danger of asphyxia. The patient has a difficulty in standing, his gait tottering; the general sensibility is diminished, the appetite normal; habitual apyrexia; no involuntary evacuations; the urine does not contain any albumen. 300 grammes of blood were drawn.

*Analysis of 1000 parts of blood.*

| | | |
|---|---|---|
| Water | | 774·792 |
| Globules | | 165·892 |
| Fibrin | | 1·443 |
| Solid matters of the serum | organic | 47·543 |
| | inorganic | 10·330 |

*Enfeeblement of the intellectual faculties—General paralysis—The excretions occasionally involuntary—Absence of ambitious monomania—Agitation—Considerable diminution of tactile sensibility—Alternation of spasmodic shocks and attacks of catalepsy—Considerable diminution of the globules—Augmentation of the water—The fibrin and the solid matters of the serum retaining their normal limits.*

CASE 6.—Henin, a locksmith, 40 years of age; has been at Bicêtre since the year 1843; is of a robust constitution, somewhat stout, has brown hair, black eyes, and a florid complexion.

At the present time, (10th of May, 1847,) the patient presents the following condition. He experienced considerable difficulty in his pronunciation; great embarrassment in the motions of his upper and lower limbs; stands insecurely, and totters in walking. He is, however, distinguished by great physical activity, is almost constantly pacing up and down the wards, and employs himself frequently in sweeping the court. He is frequently agitated during the night, gets up, tries to dress himself, screams out, and it is then necessary to secure him to his seat, or to put on a strait-waistcoat. The sphincter of the anus is occasionally incapable of retaining the fæcal matter. Considerable and equal diminution of sensibility in the two sides of the body; the flesh may be pinched or pricked without the patient being aware of it.

The intellectual faculties are deeply affected; the attention is weakened: the memory, which can embrace facts of events long since past, is completely null when an attempt is made to fix it on an idea of recent origin. The patient replies correctly to all questions asked regarding his name, age, and condition; but does not know where he is, and cannot say how long he has been at Bicêtre. He does not know how many meals he partakes of in the course of the day, nor does he remember what he has eaten at each meal. He has no delirious hallucinations, and has no fixed ideas with regard to fortune or dignities.

He has a tolerable appetite, although he has not eaten much for some time past.

On the 12th of May, the patient was seized with convulsive attacks; his jaws were tightly compressed, the muscles of the body were agitated by motions recurring regularly every second; the balls of the eyes were turned up, leaving only a small portion of the pupil visible; his hands were clenched, and the thumbs bent in under the fore-fingers; the mouth was not drawn to either side, and there was no foam about the lips; his arms and fingers retained every position in which they were placed. The patient appeared insensible to the influence of all surrounding objects. Apyrexia. No *bruit de souffle.* The face had not lost its ordinary colour.

One hundred grammes of blood were drawn during one of these attacks.

*Analysis of* 1000 *parts of blood.*

| | |
|---|---|
| Water | 879·020 |
| Globules | 32·847 |
| Fibrin | 2·990 |
| Solid matters of the serum | 85·143 |

The enormous diminution of the number representing the globules, the most considerable that we have as yet met with, may be explained at least in part, by the insufficient food taken by the patient for some time past. This diminution coincides alternately with the manifestation of convulsive shocks, and the cataleptic immobility. The face retained its usual colour during these attacks—a very remarkable phenomenon, to which M. Andral has already drawn attention in proof of the inefficiency occasionally manifested by the capillaries to betray the most decided anæmia.

*Insanity—Paralysis of motion and of sensation—Absence of ambitious monomania—Decided tendency to cerebral congestion—Very slight diminution of the globules—Diminution of the fibrin.*

Case 7.—Dardel, a mason, aged 44, of a strong constitution and sanguineous temperament, and some degree of embonpoint. Entered

Bicêtre in the month of January, 1847, since which time he has exhibited several alternations of amendment and relapses.

At the present time (15th of August, 1847) he presents the following condition:—He answers with slowness and difficulty the questions addressed to him; remembers neither his name, age, nor occupation; does not know how long he has been at Bicêtre; has no very evident ambitious hallucinations; a constant tendency to drowsiness.

There is complete insensibility of the skin, even when pinched, or deeply pricked; decided embarrassment of pronunciation; a trembling of the lips in speaking; incapacity of walking during the last few days; the face retains its usual colour; the pulse seventy-eight in the minute, moderately strong; the skin is cool. The patient has but little appetite, and has only eaten two basins of soup a day during the last two months. One hundred grammes of blood were drawn.

*Analysis of 1000 parts of blood.*

| | |
|---|---|
| Water | 800·662 |
| Globules | 107·903 |
| Fibrin | 1·920 |
| Solid matters of the serum | 89·515 |

The want of appetite and the insufficiency of food cause the globules to be slightly reduced below their normal limits. The most interesting fact to be noticed, however, is, that a very marked tendency to cerebral congestion coincides with the diminution of the fibrin.

*Misery and distress—Hallucinations and acute mania—Cure—Proud and ambitious monomania—Paralysis of motion and sensation—Contraction of the fingers—Illusions of the senses without any enfeeblement of the intellectual faculties—Voracity—Diminution of the globules—Slight augmentation of the albumen.*

Case 8.—Fouilloud entered the Hotel Dieu on the 5th of April, 1847, and occupied No. 44, in the ward St. Lazare, that of M. Martin Solon. He was a bootmaker by trade, aged 51, of a worn-out constitution, and with the appearance of a lymphatic temperament. He is not aware that madness has existed in his family. Has always been in good health up to the year 1841. At this period he was suddenly seized by an attack of maniacal excitement, in consequence of domestic troubles and a deep-seated grief. He then began to see fantastic figures flit before him; as for instance, a black man having horns, and a tail, from which sparks were emitted; he soon became furious, broke all the furniture of his shop and threw it out of the window. He was then admitted into Bicêtre, in the ward of Mons. Leuret. Continued to have numerous hallucinations, and often fancied men's voices were addressing him, which seemed to come from persons concealed at the head of his bed, who generally muttered, *Fouilloud, tu es f*——. He would turn his head round, but could see no one. While M. Leuret was at his bedside making his usual round, he heard the same voices utter the words, *Fouilloud, tu es fou.* He believed for a long time that the pupils who accompanied M. Leuret amused themselves at his expense by using these terms to him, and he would then reply, *Il faut des fous pour amuser les imbécilles.*

He left Bicêtre, nearly cured, at the end of a month, but returned in 1844, for the same species of insanity. On his second admittance, an ambitious and proud monomania predominated over all his other hallucinations, and he then believed himself possessed of five millions, to have been created Prince of Moskowa, and to have received Siberia as an appanage from the Emperor of Russia. He left Bicêtre for the second time, on the 27th of November, 1844, notwithstanding the advice of M. Leuret, who justly considered that he was not in a fit condition to leave the Hospital. He even then exhibited a slight feebleness in his legs, and walked with difficulty, inclining his body slightly to the right.

His condition on the 25th of April, 1847, was as follows:—Considerable diminution of sensibility in the skin of the face, and of the upper and lower limbs of the body on both sides. The patient does not feel being pinched or pricked at these parts. The corresponding weakness of mobility is somewhat greater on the right than on the left side; great difficulty in walking and standing; the patient can, however, raise his arms and legs in his bed without any very notable appearance of effort. The convulsion of the muscles presiding over phonation is so great as to produce the most intense stammering; there is a slight contraction of the thumbs. No paralysis of the rectum or bladder. The patient has illusions of the senses on looking attentively at any object, although this is only the case when he looks at it for several minutes together. The cup containing his drink, or that destined for the patient in the neighbouring bed, frequently appears to him to be a death's head, the hollow eyeballs of which flash fire. With the exception of this disturbance of the sensorial functions, the intelligence is in a normal condition. His memory has lost none of its vivacity, and his speech is coherent. The patient analyzes his disordered condition with clearness and precision; he is quite conscious that he is insane. With respect to the sensorial delirium, which is the only psychical symptom that exists, it may be said that he appreciates his insanity justly, whilst he attributes the cause to a vitiated condition of his imagination.

The patient has not been bled for four years, has not had any hæmorrhoidal discharge for twelve years. He has a keen appetite, and would gladly eat double the quantity of his allowance. Apyrexia. Tolerably firm pulse. No excess in the perspiration, urine, or fæces.

On the 26th of April, 100 grammes of blood were drawn.

*Analysis of* 1000 *parts of blood.*

| | | |
|---|---|---|
| Water | | 795·70 |
| Globules | | 95.50 |
| Fibrin | | 3·04 |
| Solid matters of the serum | organic | 95·30 |
| | inorganic | 10·46 |

(*To be concluded in our next.*)

